# High-resolution profiling of bacterial transcriptomes by SEnd-seq

**DOI:** 10.1042/BCJ20250251

**Published:** 2026-08-19

**Authors:** Xiangwu Ju, Shixin Liu

**Affiliations:** 1 Laboratory of Bacterial Gene Regulation and Drug Resistance, Shanghai Institute of Materia Medica, Chinese Academy of Sciences, Shanghai, China; 2 Laboratory of Nanoscale Biophysics and Biochemistry, The Rockefeller University, New York, NY 10065, U.S.A.

**Keywords:** Bacterial transcriptomics, Full-length RNA profiling, Gene regulation, RNA life cycle, SEnd-seq, transcription

## Abstract

Transcription—the process by which genomic DNA is converted into RNA—is a highly dynamic and tightly controlled process across all domains of life. Although bacteria were once regarded as relatively simple organisms, their transcriptomes are now recognized to be remarkably complex, heterogeneous, and subject to multilayered regulation. Despite the availability of an abundance of sequenced bacterial genomes, a comprehensive understanding of how bacteria tune their transcriptional output to adapt to changing environments remains lacking. To this end, SEnd-seq (simultaneous 5′ and 3′ end sequencing) was developed as a high-throughput approach uniquely capable of simultaneously capturing both 5′ and 3′ ends of individual RNA molecules, enabling the reconstruction of full-length transcripts. By capturing each RNA molecule as a distinct molecular entity with single-nucleotide resolution, SEnd-seq has uncovered previously unrecognized transcriptional features across diverse bacterial species, including even the well-studied *Escherichia coli*. This method performs robustly across a wide range of RNA species and organisms, including hard-to-lyse pathogens such as *Mycobacterium tuberculosis*. Moreover, SEnd-seq exhibits high sensitivity for detecting low-abundance RNA and is compatible with various target RNA enrichment strategies, as well as genetic, chemical, and functional perturbations, enabling context-specific transcriptomic analyses. As a versatile and broadly adaptable technology, SEnd-seq provides comprehensive insights into transcriptional regulation, RNA processing, and the coordination between RNA-based processes, thereby uncovering potential targets for antibiotic development. In the present review, we summarize the features and applications of SEnd-seq and discuss its future methodological development and expansion into broader biological and biomedical research contexts.

## Introduction

Bacteria represent one of the most diverse and abundant forms of life on Earth and constitute a fundamental component of all ecosystems [1]. Within the human body, bacterial cells coexist in numbers comparable to those of human cells, making extensive contributions to host nutrition, metabolism, immune development, and overall health [2]. Meanwhile, over 300 bacterial species are known to cause human disease. For example, *Mycobacterium tuberculosis* (Mtb), the etiological agent of tuberculosis, still kills more than one million people worldwide annually [3].

To survive, bacteria must rapidly adapt to changing environments largely by dynamically regulating their transcriptional output [4]. Despite the availability of thousands of sequenced genomes, bacterial transcriptomes remain incompletely characterized. In bacteria, transcription is carried out by a highly accurate and robust molecular machine called RNA polymerase (RNAP), whose activity is meticulously controlled by myriad regulatory factors [5,6]. Despite their compact genomes, bacteria harbor complex transcriptomes comprising diverse RNA species, including ribosomal RNA (rRNA), transfer RNA (tRNA), messenger RNA (mRNA), antisense RNA (asRNA), and regulatory small RNA (sRNA), many of which contain additional chemical modifications [7].

The RNA life cycle—from transcription to degradation—is tightly controlled, with information governing RNA function and regulation largely lying in the primary sequence, which in turn dictates its secondary and tertiary structures [7]. In bacteria, transcription start sites (TSSs) and transcription termination sites (TTSs) define the nascent boundaries of transcription units (TUs) and are key determinants of operon organization. Heterogeneity in TSS and TTS usage generates overlapping TUs and complex operon architectures [8,9]. Moreover, the final transcriptome is further shaped by additional regulatory mechanisms acting during and after transcription. These include transcriptional pausing, premature termination, RNA processing, and the coupling of transcription and translation, all of which influence transcript abundance, stability, and functional output [10,11]. Therefore, there is a growing need for comprehensive experimental approaches capable of extracting maximal transcript information to profile gene expression and reveal detailed regulatory mechanisms.

High-throughput transcriptome profiling approaches, particularly next-generation RNA sequencing (RNA-seq), have revolutionized our understanding of bacterial transcriptomes by providing a comprehensive characterization of gene expression patterns [12]. However, transcriptome profiling in bacteria remains inherently challenging due to the short half-life of mRNAs, rapid transcriptional changes, lack of poly(A) tail, wide variation in genomic GC content across species, the hard-to-lyse bacterial wall for some species, and the broad dynamic range of transcript abundance [13,14], all of which complicate the accurate quantification of gene expression [15]. Moreover, strand fragmentation, which is typically required in standard RNA-seq workflows for bacteria, disrupts native full-length transcript information [15], making it difficult to accurately define TSSs, TTSs, operon structures, and isoform diversity. In addition, many RNA-seq-based approaches do not distinguish between nascent RNA and processed RNA intermediates [13]. These limitations have driven the development of terminus-specific sequencing technologies designed to capture the RNA ends at high resolution (Table 1). Methods such as 5′ rapid amplification of cDNA ends (5′ RACE) [16], Term-seq [17], and differential RNA sequencing (dRNA-seq) [18] allow more precise mapping of transcript boundaries at either the 5′ end or the 3′ end. Rend-seq (end-enriched RNA sequencing) can map both ends with single-nucleotide resolution but does so without correlating the two termini [19]. Transcript isoform sequencing (TIF-seq) enables the simultaneous mapping of both boundaries in individual molecules via bridge ligation-mediated circularization of RNA-derived double-stranded DNA; however, this method faces challenges regarding circularization efficiency and the coverage of long transcripts [20,21]. Single-molecule real-time-Cappable-seq (SMRT-Cappable-seq) utilizes third-generation single-molecule long-read sequencing to profile full-length transcripts but relies on transcript size selection during library preparation, resulting in incomplete representation of the native transcriptome [22]; furthermore, its sequencing throughput is lower than that of second-generation platforms such as Illumina, limiting its scalability [15,23].

**Table 1:** Comparison of terminus-profiling RNA-seq methods

Method	Primary utility	Resolution	End correlation	Platform	Key strength/limitation
*Standard RNA-seq*	Global gene expression profiling	Low	None (ends obscured)	2nd Gen	High throughput; lacks precise boundary information
*5*′ *RACE/dRNA-seq*	Mapping TSSs	High	5′ end only	2nd Gen	High 5′ sensitivity; captures only one end
*Term-seq*	Mapping TTSs	High	3′ end only	2nd Gen	High 3′ sensitivity; captures only one end
*Rend-seq*	Mapping both 5′ and 3′ ends	High	Unlinked (fragmentation-based)	2nd Gen	Dual-end capture; uncoupled 5′ and 3′ end information
*TIF-seq*	Simultaneous 5′ & 3′ end capture	High	Linked via dsDNA circularization	2nd Gen	Coupled 5′ and 3′ end information; length bias
*SMRT-Cappable-seq*	Full-length transcript profiling	High	Linked (Long-read)	3rd Gen	Direct full-length reads; low throughput, length selection
*SEnd-seq*	Simultaneous 5′ & 3′ end capture	High	Linked (cDNA circularization)	2nd Gen	High sensitivity for low-abundance RNAs, relatively low length bias; relatively complex library prep

2nd Gen: second-generation sequencing platforms (e.g., Illumina); 3rd Gen: third-generation single-molecule sequencing platforms (e.g., PacBio, Oxford Nanopore).

SEnd-seq (simultaneous 5′ and 3′ end sequencing) was developed to enable simultaneous characterization of both the 5′ and 3′ ends of individual RNA molecules in bacteria [9]. Given the limited introns in bacterial genomes, full-length RNA sequences can be reconstructed via terminus mapping, yielding datasets comparable to those obtained with SMRT-Cappable-seq [22]. In addition, SEnd-seq is capable of profiling a broad spectrum of RNA types of varying lengths while mitigating biases arising from the broad dynamic range of gene expression. Importantly, the method performs reliably across several bacterial species tested so far, including the well-studied *Escherichia coli* (*E. coli*) and bacteria with challenging cell wall structures such as Mtb, revealing transcriptomes at nucleotide resolution and uncovering previously unrecognized transcriptional features [9,10]. In the present review, we summarize the advantages and limitations of the SEnd-seq method and discuss its current applications and future development.

## Introduction to SEnd-seq

### Experimental strategy

**Figure 1:**
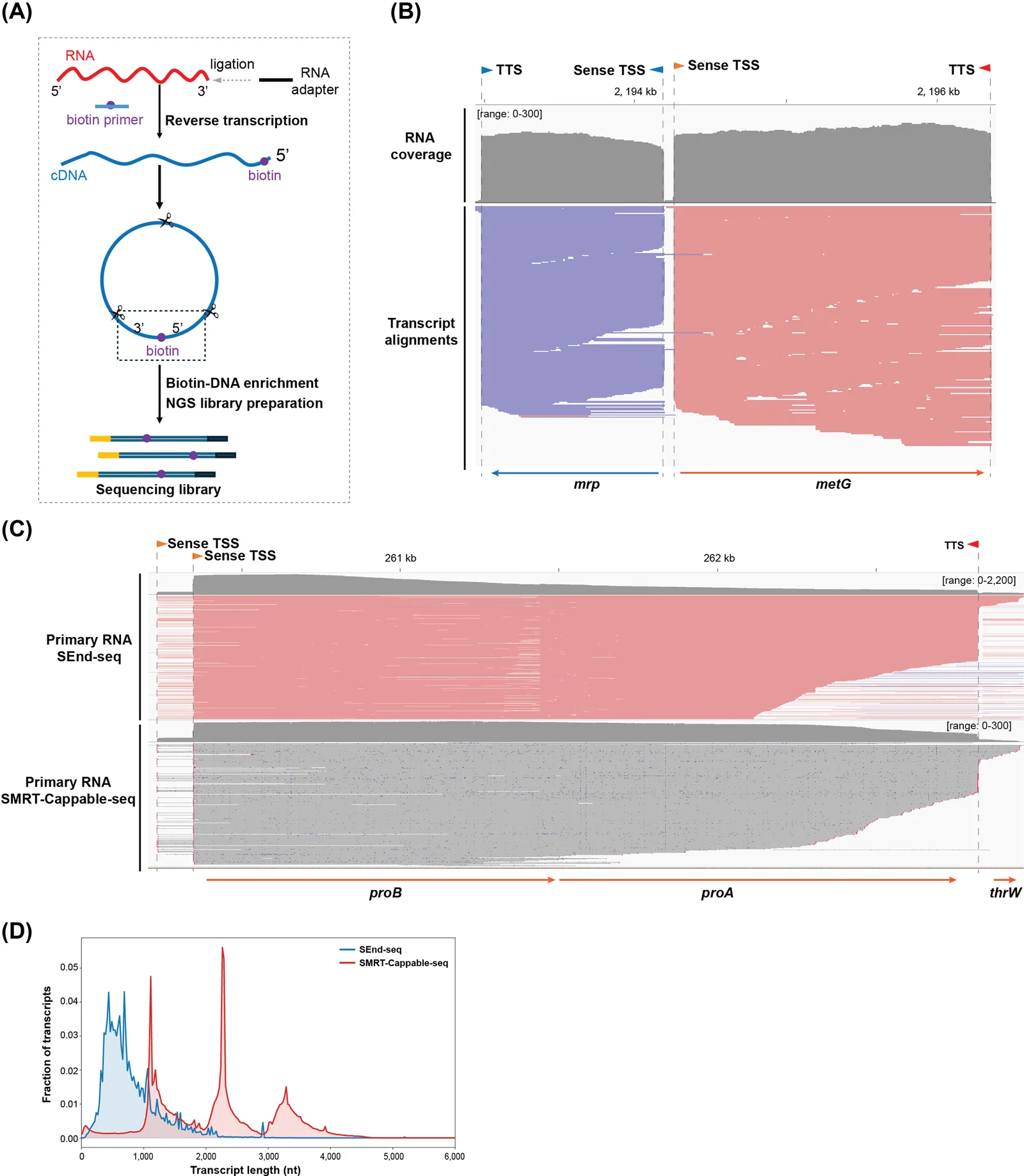
SEnd-seq method and example data (**A**) Schematic of the SEnd-seq workflow. RNA is reverse-transcribed into cDNA, which is subsequently circularized to physically link the original 5′ and 3′ termini. The circular cDNA is fragmented, and junction-containing fragments are enriched for library preparation and high-throughput sequencing. (**B**) Representative SEnd-seq track of the *E. coli* transcriptome. Data from log-phase cells show the high-resolution reconstruction of full-length transcripts. Red and blue tracks represent transcripts originating from the plus and minus strands, respectively. (**C**) Comparison of primary RNA SEnd-seq and primary RNA SMRT-Cappable-seq data at a representative locus demonstrates the high concordance between the two methods in reconstructing full-length transcripts. (**D**) Genome-wide transcript length distributions recovered by SEnd-seq and SMRT-Cappable-seq. SMRT-Cappable-seq libraries are typically size-selected into 1–2 kb, 2–3 kb, and >3 kb fractions prior to sequencing, resulting in characteristic enrichment peaks in the length distribution. Note that these datasets were generated from cells grown in different media, which could contribute to the difference in RNA length distribution.

The SEnd-seq approach was developed based on a circularization strategy for RNA-derived complementary DNA (cDNA). Following high-quality RNA extraction, which needs to be optimized for specific bacterial species, synthetic spike-in RNAs are added at a defined ratio to enable accurate normalization and quantitative comparison across samples [9]. The 3′ end of each RNA molecule is then ligated to an RNA adapter and reverse-transcribed into cDNA using a biotin-modified primer complementary to the adapter and a high-performance reverse transcriptase MarathonRT [24]. Next, TS2126 ligase I is used to join the 5′ and 3′ ends of each cDNA molecule through intramolecular ligation with minimal bias related to transcript length and terminal sequence composition [25], forming a terminus–adapter–terminus junction at the ligation site. After second-strand synthesis, DNA fragmentation, and sequencing adapter ligation, the fragments containing ligation junctions are enriched with streptavidin beads and subjected to PCR amplification and sequencing on Illumina platforms (Figure 1A). Through downstream bioinformatic analysis, the original transcript sequences are reconstructed by correlating the RNA 5′- and 3′-end information. This strategy enables the reconstruction of full-length RNA species while preserving native transcript boundaries (Figure 1B).

### Technical considerations and best practices

Successful implementation of SEnd-seq requires careful attention to technical details that directly influence data quality. Rapid quenching of bacterial cells prior to RNA extraction and minimization of RNase exposure are essential for ensuring high-integrity input RNA, which significantly improves the success rate and quality of library preparation [26]. Although rRNA depletion is optional for SEnd-seq, it can further improve sequencing depth and enhance the downstream analysis sensitivity. For pathogenic or hard-to-lyse species, such as Mtb, additional measures, such as rapid cell inactivation using guanidinium thiocyanate buffer, optimized bead-beating cell lysis, and customized rRNA depletion probes, may be necessary to faithfully capture the original RNA status [10]. The use of spike-in RNAs is strongly recommended to support accurate quantitative comparison across conditions. Notably, maximizing nucleic acid recovery at each step—achieved through optimized magnetic bead-based purification or phenol-chloroform extraction—is critical for the overall library yield and quality, given the duration and complexity of the SEnd-seq workflow. Moreover, optimization of enzyme-to-substrate ratios and reaction conditions (in particular, reverse transcription and cDNA circularization) is important for maximizing substrate recovery and library preparation efficiency [24], as excess enzymes may increase nonspecific interactions with single-stranded cDNA and affect substrate recovery during subsequent purification steps. Collectively, these practices help ensure that SEnd-seq preparation captures the native transcriptomes with high reproducibility.

## Technical advantages of SEnd-seq for bacterial transcriptomics

### Full-length RNA sequencing

The defining technical advantage of SEnd-seq lies in its ability to simultaneously capture the native 5′ and 3′ ends of individual RNA molecules. Transcript boundaries in standard RNA-seq are often obscured by RNA fragmentation and length filtering, which result in reduced signal intensity at the 5′ and 3′ termini [27]. In comparison, SEnd-seq preserves and precisely captures terminal information, enabling accurate reconstruction of full-length transcripts in a strand-specific manner [9] (Figure 1B). Consequently, the resulting transcript architectures are largely consistent with those obtained using third-generation long-read sequencing platforms [28], while retaining the high throughput, accuracy, and cost-effectiveness of second-generation short-read technologies (Figure 1C). Genome-wide transcript length analysis showed that SEnd-seq recovers a broader range of primary RNAs, particularly those shorter than 1 kb, compared with SMRT-Cappable-seq, another full-length transcript profiling method (Figure 1D). Notably, while detecting very short transcripts (<100–200 nt) remains a challenge for most RNA-seq methods, SEnd-seq alleviates the loss of smaller species by filtering only the shortest fragments (<50 nt) required to reduce adapter interference, thereby permitting the detection of a broad spectrum of RNA species. The resulting data are readily visualized using the Integrative Genomics Viewer [29], with each line representing an individual RNA molecule. These capabilities enable SEnd-seq to achieve high-sensitivity, full-length transcriptome profiling, establishing it as a versatile platform that provides a unified and quantitative framework for studying bacterial gene regulation and revealing isoform diversity and operon complexity [22].

### Transcript boundary detection with single-nucleotide resolution

Using an adapter-dependent ligation strategy, SEnd-seq is able to detect TSSs and TTSs with high confidence at nucleotide resolution. In SEnd-seq, each TSS or TTS is represented by multiple full-length transcripts rather than being identified solely from accumulated short terminal signal as in many other methods [30]. This ‘end-to-end’ design substantially reduces technical noise and enables accurate assessment of individual TSS usage and termination efficiency at specific TTSs. By quantifying RNA abundance at each nucleotide position, significant changes in signal profiles across gene bodies can indicate regulatory events such as transcriptional pausing, RNA processing, and degradation.

Furthermore, because SEnd-seq simultaneously detects the 5′ and 3′ ends of individual RNA molecules, it enables analysis of how alternative TSS usage influences downstream transcription elongation and termination. For example, we found growth condition-dependent coupling between TSS selection and downstream TTS selection in *E. coli* [9], suggesting a long-range regulatory mechanism. SEnd-seq can also be used to distinguish closely spaced or overlapping initiation events as well as overlapping termination sites, such as bidirectional terminators identified in *E. coli* [9] which were overlooked by standard RNA-seq and other single-terminus profiling methods. Together, these capabilities enable a comprehensive view of bacterial transcriptomes and precise reconstruction of TUs, uncovering novel regulatory mechanisms.

### Detection of low-abundance RNAs with high sensitivity

Another important advantage of SEnd-seq is its high sensitivity for detecting low-abundance transcripts. Because of the read-length requirements of current short-read sequencing platforms, RNA fragmentation is typically needed for standard RNA-seq [31]. As a result, sequencing depth is disproportionately consumed by long and abundant transcripts—most notably rRNAs—which generate multiple reads per transcript during fragmentation, thereby reducing sensitivity for low-abundance RNA species and necessitating rRNA depletion to profile the remaining transcriptome. In contrast, SEnd-seq captures each RNA molecule as a single intact entity, such that each paired-end read represents one full-length transcript largely independent of transcript length. Accordingly, whereas a long RNA molecule may generate many reads in standard RNA-seq, it is typically represented by a single paired-end read in SEnd-seq in the same way as a short RNA molecule. Consequently, transcript abundance in SEnd-seq datasets is quantified on a molecule basis rather than a fragment basis, substantially enhancing sensitivity and quantitative accuracy for low-abundance RNAs. Notably, even without rRNA depletion, more than 20% of SEnd-seq reads map to mRNA genes, compared with ∼1-5% in total RNA libraries generated by standard RNA-seq without rRNA depletion. This feature is particularly valuable for transcriptomic profiling of organisms that lack suitable rRNA depletion reagents.

In addition, SEnd-seq provides unambiguous strand-specific information by physically linking the 5′ and 3′ ends of each cDNA generated from the original RNA molecule in their respective orientation, rather than relying on the dUTP-based strand assignment method used in standard RNA-seq [15], which is subject to enzymatic bias and incomplete synthesis, particularly for high GC-content genomes like that of Mtb [32]. Furthermore, SEnd-seq facilitates the capture of various structured RNA species, including tRNA-derived fragments. These products are frequently lost in standard RNA-seq workflows due to their small size and the presence of secondary structures or base modifications [33]. Together, these capabilities allow SEnd-seq to reveal previously unannotated transcript features and regulatory RNA populations that are difficult to resolve using fragment-based sequencing approaches.

### Detection of primary, processed, and nascent transcripts

In bacteria, newly synthesized RNA transcripts typically carry a 5′ triphosphate group and are referred to as primary transcripts. This 5′ triphosphate can be converted to a 5′ monophosphate by enzymes such as RppH in *E. coli* [34]. Endoribonucleases can also attack RNA internally, generating processed RNAs that also bear a 5′ monophosphate [35], thereby rendering these RNAs more susceptible to RNase-mediated degradation [36]. Primary transcripts can be selectively enriched by enzymatic capping with a modified guanosine triphosphate (3′-desthiobiotin-GTP) followed by streptavidin-biotin affinity purification, enabling the generation of primary RNA SEnd-seq datasets for TSS analysis [22]. In parallel, processed RNAs bearing a 5′ monophosphate can be specifically labeled with a 5′ RNA adapter, enabling their identification and computational segregation within the total RNA SEnd-seq datasets [37]. These procedures make SEnd-seq useful for studying specific phases of the RNA life cycle such as transcription initiation and processing.

Because SEnd-seq is compatible with low-input RNA and does not require RNA fragmentation, it is therefore well suited for low-abundance protein-bound RNA populations. SEnd-seq can be readily combined with enrichment strategies for protein-RNA complexes. One successful application involves the sequencing of nascent transcripts that remain associated with RNAP by isolating the transcription complex through His-tag–based affinity purification [10,38]. RNAP-bound RNAs represent nascent transcripts, and enrichment of reads mapped within gene bodies largely reflects transcriptional intermediates associated with RNAP pausing. This approach thus provides a means to distinguish paused RNA species from terminated ones.

### Correlating transcript structure with expression profiling

While bacterial mRNA transcriptomes were traditionally viewed as collections of largely full-length RNAs, recent high-resolution mapping has revealed an abundance of truncated RNAs within gene bodies [10,39], raising the question of whether these species play a functional role in governing gene expression. Thanks to its full-length transcript profiling ability, SEnd-seq enables reproducible measurements of gene expression by contextualizing RNA signals within their complete transcript architecture. While standard RNA-seq relies on fragment-derived counts to estimate gene expression, SEnd-seq preserves the structural integrity of each individual molecule. This distinction is critical for accurately interpreting the functional transcriptome, as it permits the differential profiling of mature, full-length mRNAs and transcriptional intermediates. The preservation of transcript isoforms defined by their TSS-TTS pairs, combined with synthetic spike-in controls, facilitates a robust comparison of transcript levels across varying growth states and bacterial species [10].

Comparative analyses of genome-wide gene expression profiles generated by standard RNA-seq and SEnd-seq in *E. coli* reveal a strong correlation between the two datasets [9]. Nonetheless, the enhanced resolution of SEnd-seq becomes evident when the transcript structure is examined. For example, the incomplete transcripts identified by SEnd-seq in Mtb are characterized by an RNA abundance drop-off at 200-500 nucleotides downstream of TSSs. A corresponding drop-off can be detected in standard RNA-seq data but is substantially obscured. Thus, while standard RNA-seq captures overall expression trends, SEnd-seq provides richer information about processes that shape the transcript boundaries [9,10]. Furthermore, compared to the overall transcript abundance, the downstream RNA level correlates more strongly with the ribosome binding signal, suggesting that full-length transcription more accurately reflects functional gene expression. Therefore, whereas standard RNA-seq provides a gross measure of total RNA abundance, SEnd-seq offers a more precise representation of the functional, protein-coding transcriptome.

## Biological insights enabled by SEnd-seq analysis

### Promoter selection

Transcription initiation lies at the center of bacterial gene regulation and largely determines the level of transcriptional output [40]. Changes of TSS usage and promoter strength reflect cellular responses to environmental and physiological signals. SEnd-seq has demonstrated high sensitivity and precision in detecting TSSs in *E. coli* and Mtb [9,10]. The resulting datasets recapitulate most known TSSs while also revealing a substantial number of previously unannotated initiation sites, including weak, condition-specific, and internal promoters located within annotated genes. Notably, SEnd-seq robustly detects leaderless TSSs across different bacterial species, highlighting the prevalence of non-canonical translation initiation in bacteria. A recent study analyzed the SEnd-seq TSS datasets and found the conserved promoter architecture in both *E. coli* and Mtb [41]—for example, the −10 motif (TANNNT) recognized by the housekeeping sigma factor (σ^70^ for *E. coli* and σ^A^ for Mtb) [42]—thus validating these annotations. The TSSs can be further categorized based on their genomic locations and transcriptional orientations—classified as primary, internal, and antisense TSSs—revealing the remarkable complexity and heterogeneity of the bacterial promoter landscape.

By integrating high-confidence TSSs with reconstructed full-length transcripts, SEnd-seq further enables the establishment of promoter–operon relationships, including the identification of those TSSs that mainly drive downstream gene expression within operons. This integrated data is particularly useful for understanding regulatory mechanisms operating at the 5′ untranslated regions (5′ UTRs), such as ribosome-binding site selection and small-RNA-mediated regulation that targets 5′ UTRs to control translation efficiency [43,44]. This information is also valuable for functional genetics and targeted perturbation experiments (e.g., CRISPRi), as it helps distinguish primary promoters from internal initiation sites. By accurately mapping these boundaries, researchers can minimize polar effects on downstream operon members and achieve more precise single-guide RNA target selection.

SEnd-seq reveals extensive overlap between TSSs and TTSs of neighboring genes, as well as widespread bidirectional promoter activity [9], further highlighting the complexity of bacterial transcriptomic organization. Collectively, these findings establish SEnd-seq as a powerful platform for dissecting bacterial promoter architecture and transcription initiation landscape.

### Transcription termination

Precise transcription termination is essential for proper gene regulation, as failure to terminate transcription leads to disruption of downstream gene expression, uncontrolled transcriptional collisions, and compromised genome stability [45]. In bacteria, transcription termination is mediated mainly by two pathways: intrinsic termination, which relies on RNA secondary structures and U-rich sequences; and factor-dependent termination, most commonly Rho-dependent termination, which requires the ATP-dependent helicase Rho [46]. Regulated termination events can lead to transcriptional attenuation between upstream genes and downstream genes within an operon, a widespread mechanism that shapes operon architecture and condition-dependent gene expression. Importantly, the relative contribution of these mechanisms varies across bacterial species. SEnd-seq provides a means for genome-wide identification and classification of RNA 3′ termini. While these 3′ ends frequently correspond to TTSs, it is important to note that they may also represent stable RNA processing intermediates or prolonged RNAP pausing sites [10]. By capturing intact RNA molecules with their respective 3′ boundaries, SEnd-seq reveals sharp and reproducible signal drop-offs at transcript 3′ ends, enabling high-resolution mapping of major termination sites across the genome. Combining SEnd-seq with chemical perturbations, such as Rho inhibition using bicyclomycin in *E. coli*, further allows discrimination between intrinsic and Rho-dependent terminators [9]. Intriguingly, in Mtb, relatively few consensus TTSs, defined as sites associated with a sharp reduction in RNA read coverage at the end of TUs, were detected compared with *E. coli*, and knockdown of Rho did not cause widespread transcriptional readthrough [10]. These findings suggest that the termination mechanisms in Mtb differ substantially from those in model bacteria and await further investigation [10].

In *E. coli*, beyond intrinsic and Rho-dependent terminators, SEnd-seq has uncovered widespread bidirectional terminators positioned between opposing gene pairs, where high termination efficiency is largely independent of the canonical intrinsic terminator structure and Rho activity. Rather, convergent transcription is a key driver of efficient transcription termination [9,47]. These transcriptome-wide observations were further validated by single-molecule and structural assays, establishing a mechanistic link between RNAP collisions, RNA stem-loop structure, and termination outcome [47,48].

### Transcriptional pausing and elongation dynamics

Given transcription's central role in bacterial gene regulation, different bacterial species have evolved distinct strategies to control their transcriptional output [41,49]. Beyond transcription initiation and termination, SEnd-seq can also be used to monitor transcription elongation dynamics by capturing the intermediate RNA species whose 3′ ends are located before gene ends. This allows genome-wide mapping of pause sites and their associated sequence motifs, providing mechanistic insight into elongation control. To quantify these elongation dynamics, we have used a numerical ‘progression factor’ (PF). This phenomenological metric is defined as the ratio between average RNA coverage in the downstream region (from 500 nucleotides downstream of the TSS to the gene end) and that in the upstream region (first 200 nucleotides downstream of the TSS) [10]. In principle, any process that reduces the RNA abundance toward the gene end—such as premature transcription termination, extended pausing, or degradation—can result in a low PF. As described in recent kinetic modeling of bacterial transcription, the PF value is influenced by the relative rates of elongation, termination, and RNA decay [50].

In *E. coli*, the RNA coverage across gene bodies remains largely constant. In contrast, the Mtb transcriptome exhibits markedly different features. SEnd-seq revealed unexpectedly pervasive incomplete transcripts originating from TSSs, resulting in low PF values for most Mtb genes. These RNAs may represent paused or prematurely terminated transcription products. When further enriched for RNAP-associated transcripts, the SEnd-seq data showed that these incomplete RNAs are predominantly bound to RNAP, supporting the model that they are generated primarily by transcriptional pausing rather than premature termination or RNA degradation [10]. These findings depict a mycobacterial transcriptome in which paused transcription is central to the regulation of transcription elongation. This unique pausing pattern is likely adaptable to environmental changes and may be exploited for novel antibiotic development.

### RNA processing and degradation

Another strength of SEnd-seq is its ability to systematically characterize RNA processing and degradation pathways by resolving both 5′ and 3′ ends of processed intermediates. RNA processing, maturation, and RNase degradation events can be deduced from RNA end mapping, such as the 5′ end cleavage by RNase P and 3′ end cleavage by RNase E that are involved in *ssrA* maturation in *E. coli* [9]. Similarly, processing events can be observed in rRNA and tRNA maturation. When coupled with genetic perturbation of RNase activity—such as CRISPRi-mediated knockdown of individual RNase genes—SEnd-seq offers a robust strategy for dissecting specific RNase activities across entire bacterial genomes. Given the increasing evidence implicating RNases in bacterial stress adaptation and antibiotic resistance [51], such data are expected to provide important insights into antibiotic resistance mechanism. Moreover, SEnd-seq can be integrated with transcriptional inhibition through high-dose rifampicin treatment followed by time-course sampling, enabling quantitative measurement of RNA decay kinetics, including degradation rates, decay direction, and gene-to-gene variability, thereby linking RNA turnover dynamics to transcriptional output.

### Non-coding RNA profiling

**Figure 2:**
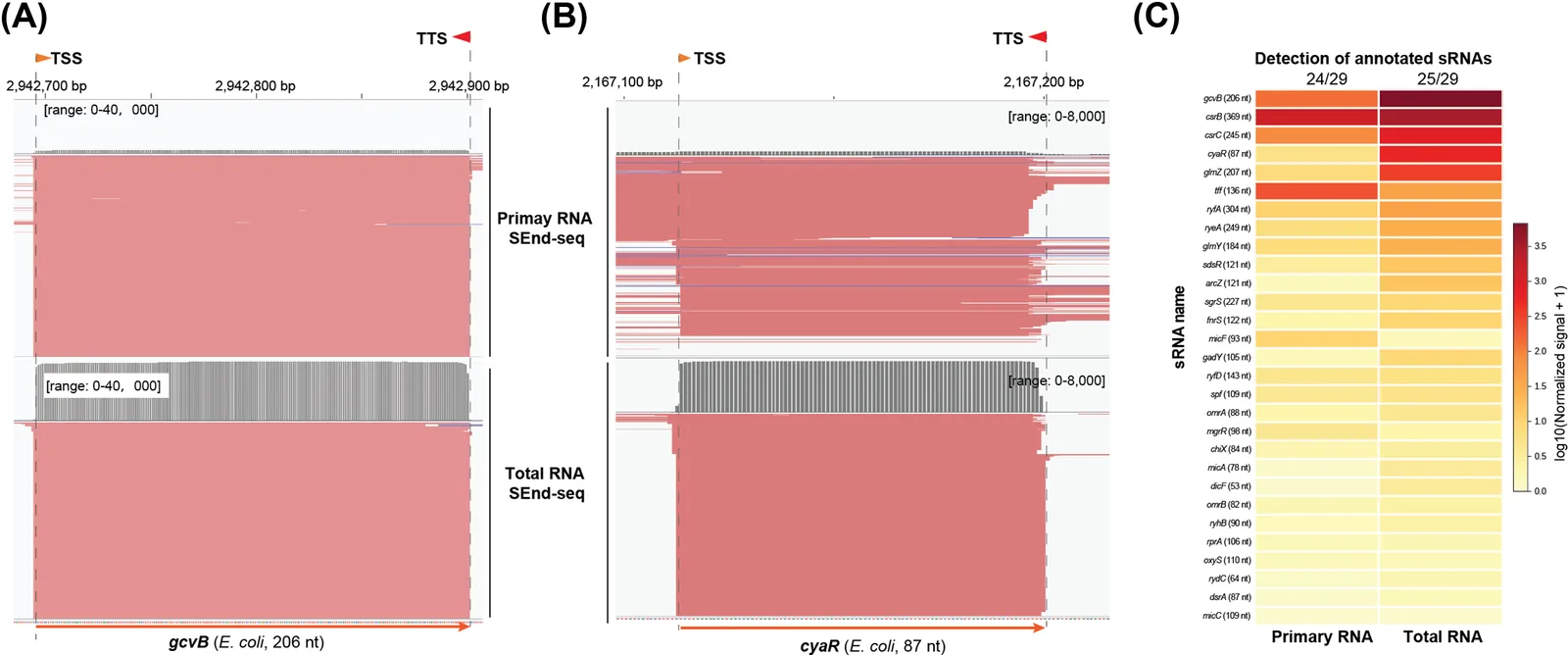
Detection of annotated *E. coli* sRNAs by SEnd-seq (**A,B**) Representative SEnd-seq track of two annotated *E. coli* sRNAs (*gcvB* and *cyaR*) detected by SEnd-seq. Primary RNA and total RNA SEnd-seq datasets generated from logarithmic-phase cells demonstrate high-resolution identification of both 5′ and 3′ transcript boundaries. (**C**) Heatmap showing the normalized SEnd-seq signal of annotated *E. coli* sRNAs in the primary and total RNA datasets. The annotated sRNAs were obtained from the *E. coli* gene annotation files (NCBI, NC_000913.3). The vast majority of annotated sRNAs showed detectable SEnd-seq signals in both datasets under the growth conditions examined.

Beyond the analysis of major RNA species in the cell, SEnd-seq provides a versatile platform for investigating the biogenesis of non-coding RNAs. SEnd-seq captures sRNAs, asRNAs, and other non-coding transcripts with high sensitivity, resolving not only their genomic positions but also their 5′ and 3′ boundaries (Figure 2). The relatively low expression levels of many annotated sRNAs in the current SEnd-seq datasets are likely attributable to the logarithmic growth conditions used in this study, as many bacterial sRNAs are induced primarily under specific environmental or stress conditions [52]. This analysis also reveals the abundance and orientation of asRNAs, which may contribute to transcriptional interference, promoter occlusion, and the fine-tuning of gene expression [53].

### Transcription-translation coupling

**Figure 3:**
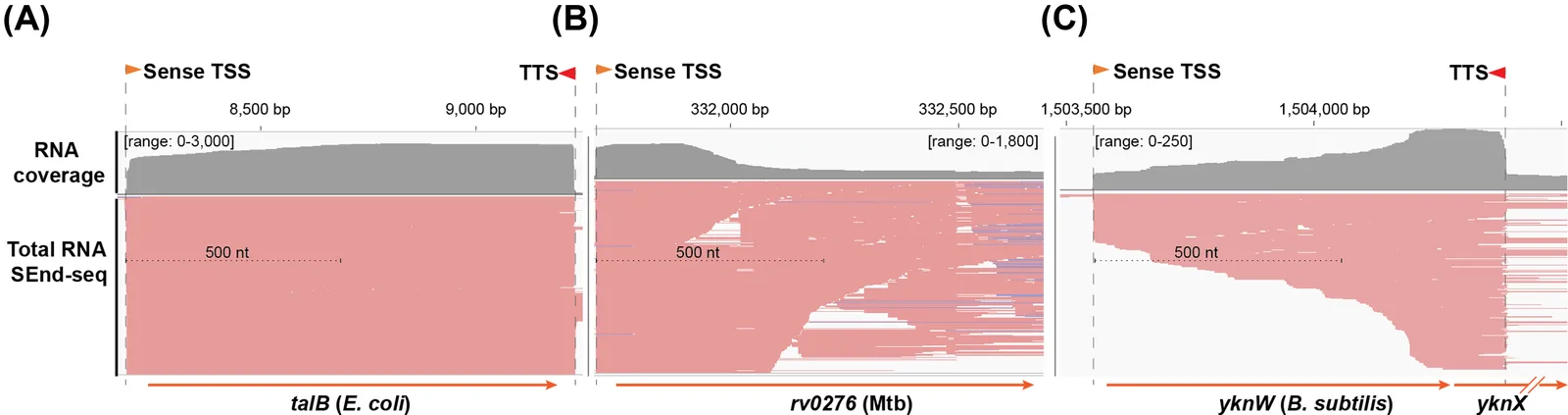
Comparative SEnd-seq analysis across bacterial species Representative SEnd-seq tracks of *E. coli* (**A**), Mtb (**B**), and *B. subtilis* (**C**) genes show species-specific variations in transcript architecture. All samples were harvested during log growth phase.

In bacteria, due to the lack of a nucleus, mRNA translation occurs co-transcriptionally [36,54]. The extent and functional importance of transcription–translation coupling vary across bacterial species [55–57]. In *E. coli*, the RNAP and ribosome translocate at comparable elongation rates across conditions [58]. If the nascent RNA fails to recruit ribosomes efficiently, or if RNAP outpaces the leading ribosome over extended distances, the exposed nascent RNA becomes a target for Rho binding, leading to premature transcription termination [55]. In SEnd-seq datasets from *E. coli*, RNA coverage along open reading frames is generally uniform within individual genes (Figure 3A), and only a fraction of genes exhibit PF values much lower than 1. Correlative analysis of ribosome profiling datasets demonstrates that reduced translation efficiency is associated with lower PF values, suggesting that active translation is essential for sustaining productive transcription elongation [50]. In contrast, SEnd-seq data from Mtb showed a pronounced decrease in RNA coverage at the regions of 200–500 nucleotides downstream of TSSs (Figure 3B). PF analysis indicates that the majority of TUs feature low PF values (mean = 0.43). These low-PF TUs again correlate with reduced ribosome-binding signals. Moreover, treating Mtb cells with linezolid—an antibiotic that inhibits translation initiation—results in a further reduction in PF values, providing additional evidence that transcription elongation in Mtb is highly dependent on tight transcription–translation coupling [10].

Notably, unpublished SEnd-seq data show that *Bacillus subtilis*, a gram-positive bacterium where RNAPs outpace ribosomes and thus transcription and translation are largely uncoupled [56], exhibits widespread accumulation of truncated RNA fragments toward the 3′ end of genes (Figure 3C). Unlike the drop-off pattern observed in Mtb—which reflects RNAP pausing—the increasing pattern in *B. subtilis* suggests that ribosome occupancy serves to protect transcripts from 5′-to-3′ exonucleolytic degradation rather than directly promoting transcription elongation. This is consistent with the activity of RNase J1, which can initiate 5′-to-3′ degradation of mRNAs [59]. Thus, SEnd-seq provides the resolution needed to elucidate the coordination between transcription, translation, and RNA degradation, thereby informing species-specific strategies to control the RNA life cycle in bacteria.

## Challenges, limitations, and areas for improvement

### Current technical limitations of SEnd-seq

**Figure 4:**
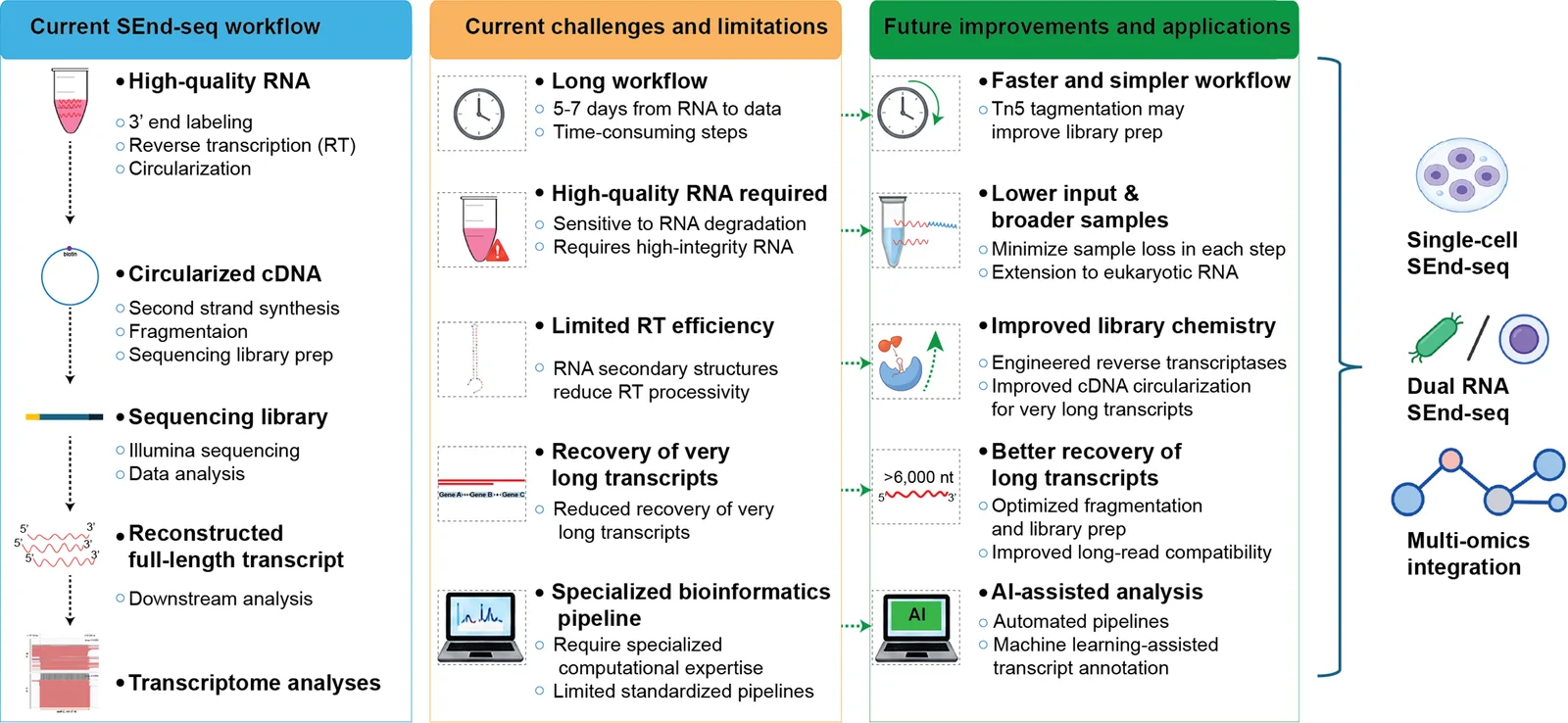
Overview of the current SEnd-seq workflow, remaining technical challenges, and future directions (Left) The current SEnd-seq workflow. (Middle) The major technical and methodological limitations of the current SEnd-seq includes its labor-intensive workflow, requirement for high-quality RNA, limited reverse transcription efficiency for structured RNAs, reduced recovery of very long transcripts, and the need for specialized bioinformatics analysis. (Right) Potential future improvements, including streamlined library preparation, reduced input requirements, improved library reaction conditions and transcript recovery, AI-assisted data analysis, and emerging applications such as single-cell SEnd-seq, dual RNA SEnd-seq, and multi-omics integration.

One of the major challenges of the current version of SEnd-seq is its complex and time-consuming workflow. Typically, the preparation of a single batch of libraries can take up to a week. Overall performance is highly dependent on the strict avoidance of RNase exposure, technical consistency, and researcher experience. The current version of SEnd-seq faces several methodological limitations. The method requires high-quality, intact RNA to preserve native transcript boundaries, meaning that samples that have undergone RNA degradation—such as those lacking immediate cell quenching or preservation—may exhibit reduced boundary resolution or distorted transcript representation. Obtaining complete reads from very long transcripts, such as those exceeding the typical operon length of 5–7 kb, remains challenging, particularly for high GC-content genomes. In addition, while SEnd-seq can effectively capture structured RNA species that are often lost in fragmentation-based libraries, extremely stable secondary structures in high GC-content genomes can still pose physical barriers to reverse transcription or circularization, leading to the underrepresentation of specific RNA classes. These inherent constraints underscore the need for careful protocol optimization, appropriate experimental controls, and cautious data interpretation when applying SEnd-seq to complex samples (Figure 4).

Future technological developments are expected to address many of these limitations. Developments should focus on reducing input RNA requirements and enhancing compatibility with partially degraded RNA, for example, through more efficient RNA–adapter ligation reactions, improved adapter designs, and optimized Tn5-based tagmentation strategies. Such improvements would extend the applicability of SEnd-seq to challenging sample types, including low-input bacterial samples. Additional opportunities include improving reverse transcription efficiency across genomes with extreme GC content and mitigating barriers imposed by RNA secondary structures, both of which currently limit uniform transcript recovery (Figure 4).

### Computational analysis pipeline

SEnd-seq data analysis requires customized species-specific bioinformatic pipelines tailored to bacterial genome architecture and regulatory features. Initial processing typically includes adapter trimming and alignment using programs optimized for SEnd-seq strategy. Downstream analyses require substantial customized coding, particularly when dealing with differences in genome size and chromosome or plasmid number. High-resolution 5′ and 3′ end calling is then performed to identify TSSs and TTSs, followed by full-length transcript reconstruction to delineate operon structures and alternative isoforms. TUs are inferred based on contiguous RNA coverage and shared boundary features, while transcription elongation analysis is based on relative RNA abundance across individual TUs.

Currently for standard RNA-seq, gene expression level is typically estimated from summed RNA signals. However, whether partial signals—such as those derived from incomplete transcripts—accurately reflect true gene expression levels remains an open question. The development of analytical frameworks that explicitly account for incomplete or intermediate RNA species would therefore improve the interpretation of SEnd-seq data and enhance its quantitative utility in gene expression analysis.

Beyond standalone transcriptome analysis, high-resolution SEnd-seq datasets can be integrated with other omics approaches, including RNAP chromatin immunoprecipitation sequencing for mapping polymerase occupancy and ribosome profiling for probing translation activity [10]. Such integrative multi-omics analysis enables a systems-level view of bacterial gene regulation.

## Future directions

### Integrating machine learning into SEnd-seq data analysis

Machine learning (ML) has demonstrated broad applicability across biological research, but its performance critically depends on the availability of high-quality datasets for model training, which are essential for achieving robust, accurate, and generalizable results [60–62]. The increasing availability of high-resolution SEnd-seq datasets therefore presents an opportunity for integrating ML approaches into transcriptomic analysis and mechanistic modeling [60]. Models trained on SEnd-seq data could be used to predict previously unannotated TSSs and TTSs [63]. Furthermore, ML models could be trained to identify the *cis*-regulatory determinants of transcription elongation, such as specific DNA sequence motifs and RNA secondary structures that trigger RNAP pausing or termination. For studies involving multiple species or experimental conditions, ML may facilitate automated transcript reconstruction and feature extraction across multiplexed datasets, improving scalability and accelerating functional annotation.

### Toward low-input and single-cell bacterial transcriptomics

A long-standing challenge in bacterial transcriptomic studies is the development of low-input or even single-cell RNA-seq methods [64], which will enable investigation of transcriptional heterogeneity and dynamic metabolic-state transitions within bacterial populations. Given its high sensitivity for detecting low-abundance RNAs, SEnd-seq may be adapted to low-input and potentially single-cell applications after the implementation of proper cell separation and RNA barcoding strategies. Such advances will extend the application of SEnd-seq to rare cell populations, such as slow-growing pathogens in host cells and environmental samples with limited biomass. Achieving single-cell SEnd-seq is expected to provide unprecedented insight into transcriptional heterogeneity, lineage-specific adaptation, and stochastic gene expression, thereby advancing our understanding of bacterial physiology at single-cell resolution and potentially elucidating the mechanisms of antibiotic resistance and tolerance.

### Extending SEnd-seq to eukaryotic transcriptomes

Although SEnd-seq was originally developed for bacterial transcriptomics, its ability to simultaneously capture and correlate native 5′ and 3′ transcript ends also makes it a desirable approach to characterizing eukaryotic transcriptomes. Adapting SEnd-seq to eukaryotic systems, however, poses additional technical challenges. Unlike in bacteria, most eukaryotic mRNAs carry a 7-methylguanosine (m^7^G) cap at the 5′ end and a 3′ poly(A) tail and frequently undergo alternative splicing. These features necessitate substantial methodological adaptation, such as the use of anchored oligo(dT)-VN primers to capture 3′ poly(A) sites and increased sequencing read length to enable the precise annotation of gene ends and reconstruction of full-length transcript isoforms, although the presence of introns remains a major complicating factor. Additional considerations include optimizing reverse transcription and circularization efficiency for long transcripts and developing workflows compatible with capped RNA species to enable accurate TSS detection.

Even with such improvements, complete reconstruction of long, intron-rich isoforms using short-read sequencing platforms may still be out of reach. Nevertheless, the unique strength of SEnd-seq in detecting both ends at the same time could still provide valuable information about alternative usage of promoters and polyadenylation sites, gene fusion events, and transcriptional interference. Moreover, long non-coding RNAs, which often exhibit limited isoform diversity and play critical roles in eukaryotic gene regulation, represent a particularly attractive target for SEnd-seq profiling, as concurrent 5′ and 3′ end mapping will substantially improve their annotation and functional characterization [65]. Therefore, SEnd-seq has the potential to become a valuable addition to the eukaryotic transcriptomics toolbox for profiling gene regulation in complex cellular systems.

### Applications in host–pathogen interactions: toward dual SEnd-seq

Simultaneous profiling of transcriptomic responses in both pathogen and host can facilitate our understanding of host–pathogen interactions. However, because pathogen-derived RNAs often constitute only a small fraction (typically <5%) of total cellular RNA, it remains challenging to profile host and pathogen transcriptomes concurrently using conventional approaches [66]. SEnd-seq can in principle be applied to infected host cells to resolve pathogen-specific transcriptional states during infection through the development of ‘dual SEnd-seq’. For example, in an Mtb-infected macrophage model, SEnd-seq could resolve the distinct 5′ and 3′ boundaries of the mycobacterial transcriptome directly from the infected host environment, potentially identifying antisense-mediated interference or other regulatory RNA interactions that occur during intracellular survival. Owing to its high sensitivity for detecting low-abundance transcripts and diverse RNA species, SEnd-seq may enable simultaneous profiling of host and pathogen transcriptomes without additional enrichment steps that could distort transcriptomic profiles [67]. This strategy will reveal infection-induced transcriptional changes of Mtb within the host, uncovering regulatory architectures—such as precise transcript boundaries and antisense overlaps within virulence loci—that are often masked in standard RNA-seq. Ultimately, dual SEnd-seq holds promise to uncover the mechanisms governing pathogen adaptation, persistence, and virulence within the host niche, informing the development of targeted therapeutic strategies.

## Conclusion

SEnd-seq has emerged as a powerful technology that substantially expands our ability to profile bacterial transcriptomes with comprehensive coverage and high resolution. By capturing the native 5′ and 3′ ends of full-length RNA molecules, SEnd-seq overcomes long-standing limitations of fragmentation-based methods and provides new insight into transcription initiation, termination, elongation dynamics, RNA processing, and operon architecture. Its applications have already advanced our understanding of bacterial gene regulation, revealing species-specific regulatory mechanisms, such as pervasive transcriptional pausing in Mtb.

Looking forward, continued methodological optimization—together with the integration of ML–based analyses, single-cell technologies, and dual SEnd-seq applications—promises to transform SEnd-seq into an even more versatile tool. As SEnd-seq can be applied across diverse bacterial species and experimental contexts, it is poised to play a prominent role in bridging fundamental bacterial physiology with translational applications, including antimicrobial development and precision medicine.

## Data Availability

The newly generated analyses presented in the present review were derived from previously published SEnd-seq datasets [9,10], publicly available sequencing datasets [22], and an unpublished *B. subtilis* SEnd-seq dataset (available from the corresponding author upon reasonable request).

## Open Access

Open access for this article was enabled by the participation of Rockefeller University in an all-inclusive Read & Publish agreement with Portland Press and the Biochemical Society.

## Abbreviations

5′ RACE: 5′ rapid amplification of cDNA ends
5′ UTR: 5′ untranslated region
asRNA: antisense RNA
cDNA: complementary DNA
dRNA-seq: differential RNA sequencing
*E. coli*: *Escherichia coli*
ML: machine learning
mRNA: messenger RNA
Mtb: *Mycobacterium tuberculosis*
PF: progression factor
RNAP: RNA polymerase
RNA-seq: RNA sequencing
Rend-seq: end-enriched RNA sequencing
rRNA: ribosomal RNA
SEnd-seq: simultaneous 5′ and 3′ end sequencing
SMRT-Cappable-seq: single-molecule real-time-Cappable-seq
sRNA: small RNA
TIF-seq: transcript isoform sequencing
TSS: transcription start site
TTS: transcription termination site
tRNA: transfer RNA
TU: transcription unit
